# Overexpressing PTTG family genes predict poor prognosis in kidney renal clear cell carcinoma

**DOI:** 10.1186/s12957-021-02225-2

**Published:** 2021-04-12

**Authors:** Yonghui Gui, Xueni Liu, Chao Wang, Peng Yang

**Affiliations:** Department of Blood Transfusion, The First Affiliated Hospital of Anhui Medical University, 218 Jixi Road, Hefei, 230032 Anhui China

**Keywords:** PTTG, KIRC, Prognostic, Biomarker

## Abstract

**Background:**

Pituitary tumor transforming genes (PTTG1, PTTG2, and PTTG3P) play key roles in the pathogenesis and development of human cancers. The studies show that overexpression of the PTTG genes is associated with tumor progression and migration. However, the function of the PTTG genes in the prognostic value of kidney renal clear cell carcinoma is rarely known by people.

**Methods:**

The expression of PTTG family genes was analyzed by the ONCOMINE, Human Protein Atlas, GEPIA2, and UALCAN database. The relationship between PTTG family genes expression level and clinical indicators including prognostic data in kidney renal clear cell carcinoma was analyzed by GEPIA2, TCGA portal, and UALCAN. cBioPortal database was used to analyze the genetic mutations of differentially expressed PTTG family members. Similar genes of the PTTG family (90 in total) obtained from GEPIA2 and Metascape were used for GO enrichment to explore the interaction among similar genes. The online tools of Metascape and STRING were used for functional and pathway enrichment analysis.

**Results:**

PTTG1, 2, and 3P mRNA and protein expression upregulated in kidney renal clear cell carcinoma kidney renal clear cell carcinoma patients compared with normal tissues. And higher expression level of PTTG family genes was associated with shorter overall survival (OS) and disease-free survival (DFS). Furthermore, overexpression of the PTTG family genes had been found correlated with individual cancer stages and pathological tumor grades. In addition, 18% of mutations in the PTTG family genes were associated with short-term survival in kidney renal clear cell carcinoma patients.

**Conclusions:**

A single PTTG gene or PTTG family genes as a whole may be a potential prognostic biomarker for kidney renal clear cell carcinoma.

## Background

Renal cell carcinoma (RCC) is one of the most common malignancies in the world, accounting for 4.2% of all new cancer cases, with an estimated 73,820 new cases in the USA in 2019. The incidence and mortality of kidney cancer are now steadily increasing, especially with kidney renal clear cell carcinoma accounting for 75% of all kidney cancers [1, 2]. Kidney renal clear cell carcinoma is the most common renal cell carcinoma. However, patients with kidney renal clear cell carcinoma usually have poor prognosis due to limited biomarkers for early detection and prognosis prediction [3]. ADC value measured by 3T is associated with prognostic parameters of retinoblastoma and lung cancer [4, 5], but it is expensive. Comparatively, bioinformation mining can be quickly and freely available. Considerable efforts have been made to study the mechanism of the occurrence, development, and metastasis of kidney renal clear cell carcinoma disease. However, the molecular characterization of kidney renal clear cell carcinoma remains unknown. In order to improve prognosis, reliable biomarkers must be found to identify high-risk kidney renal clear cell carcinoma patients who need treatment and intervention.

PTTG consists of two genes with protein products PTTG1 and PTTG2, and a processed pseudogene PTTG3P, which has been described in the context of carcinogenesis [6]. Overexpression of PTTG was reported in many cancers, such as lung, gastric, kidney, pancreatic, breast cholangiocarcinoma, psoriasis, adrenocortical carcinoma, hepatocellular carcinoma, glioblastoma, esophageal squamous cell carcinoma, and prostate cancer [7–15]. Therefore, the PTTG family genes not only act as prognostic signatures but also become druggable targets for cancer therapy. Even though extensive researches have been conducted to investigate the role of PTTG family genes in human malignant tumors, the utility of the PTTG family genes for the kidney renal clear cell carcinoma diagnosis and prognosistic role of PTTG deletion in kidney renal clear cell carcinoma remains unclear.

In this paper, the association between PTTG expression and its diagnostic as well as prognostic value in kidney renal clear cell carcinoma was investigated. In addition, potential molecular pathways were revealed for kidney renal clear cell carcinoma through analyzing the gene interaction network by obtaining similar genes.

## Methods

### ONCOMINE

ONCOMINE database (www.oncomine.org) is an integrated online cancer microarray database used for DNA or RNA sequence analysis and for facilitating gene expression analysis and discovery [16]. In this study, transcriptional expressions of PTTG members among different cancer tissues and their corresponding adjacent normal control samples were obtained from ONCOMINE database. Difference of transcriptional expression was compared by Students’ *t* test. The cut-off values for *p* value and fold change are as follows: *p* value, 0.05; fold change, 1.5; gene rank, 10%; data type, mRNA.

### UALCAN

UALCAN (http://ualcan.path.uab.edu) is an interactive web resource based on level 3 RNA sequences and clinical data from 31 cancers in the TCGA database. It is mainly used to analyze the relative transcriptional expression of a gene between tumor and normal samples, and the correlation between transcriptional expression and related clinicopathological parameters [17]. In this study, UALCAN was used to analyze the mRNA expressions of PTTG family members in kidney renal clear cell carcinoma tissues, as well as their association with clinicopathologic parameters and tumor grades, as well as individual cancer stages. The cutoff of *p* value was set as 0.01 in the Student’s *t* test.

### Human Protein Atlas

The Human Protein Atlas (https://www.proteinatlas.org) includes nearly 20 kinds of immunohistochemical expression data of common types of cancer, in which each tumor type includes 12 individual tumor sites [18]. Users can identify proteins which are differentially expressed in a particular tumor type. In this study, the protein expression of different members of the PTTG family in normal tissues and in kidney renal clear cell carcinoma tissues was compared.

### cBioPortal

cBioPortal (http://www.cbioportal.org) is an online access database used to explore cancer genomic data from multiple perspectives [19]. The gene mutation and survival data derive from 510 kidney renal clear cell carcinoma samples in the TCGA database in cBioPortal. The z-score threshold of mRNA Expression (RNASeq V2 RSEM) set as ±1.8 was also applied to explore the relationship among the genetic alterations in PTTG family members and the overall survival of kidney renal clear cell carcinoma patients. *P* value was accepted when it is less than 0.05.

### TCGAportal

TCGAportal (www.tcgaportal.org) is an online portal allowing parallel comparisons of multiple tumors and detailed analysis of individual tumors, which was used to view survival information in the PTTG family and cross-verify with other sites.

### GEPIA2

GEPIA2 (http://gepia2.cancer-pku.cn/#index) is an open access dataset used to analyze RNA sequencing expression data from 9736 tumors and 8587 normal samples of TCGA. In this study, the top 30 genes which were similar to the PTTG family in kidney renal clear cell carcinoma were selected by using the similar gene detection module. After removing the repeated genes, 90 genes were reserved for further analysis. The cutoff of *p* value was set as 0.05 in Student’s *t* test.

### Metascape

Metascape (http://metascape.org/gp/index.html#/main/ step1) is a database which collects data from more than 40 independent knowledge bases combined with rich features, interaction analysis, gene annotation, and member search. Additionally, it facilitates the comparative analysis of multiple independent and orthogonal experiments across data sets by the portal [20]. The GO module can be used to analyze the functional roles of genes related to PTTG family members in biological processes (BP), cellular components (CC), and molecular functions (MF), and KEGG pathways of the PTTG family members.

### STRING

STRING (http://string-db.org) is a database that collects, aggregates, and scores publicly available data to explore potential protein interaction networks. PPI network was generated by STRING from members of the PTTG family genes and their similar genes.

### Statistical analysis

Related statistical analysis about the correlation between mRNA expression of PTTG family members and individuals’ survival status with kidney renal clear cell carcinoma was conducted by SPSS (version 23.0). The meaningful parameters were reserved when patients’ clinicopathological parameters and mRNA expression of PTTGs had a significant correlation (*p*<0.05) for further multivariate analysis. *P* value was accepted when it is less than 0.05.

## Result

### The mRNA and protein of the PTTG family gene is overexpressed in patients with kidney renal clear cell carcinoma disease

**Fig. 1:**
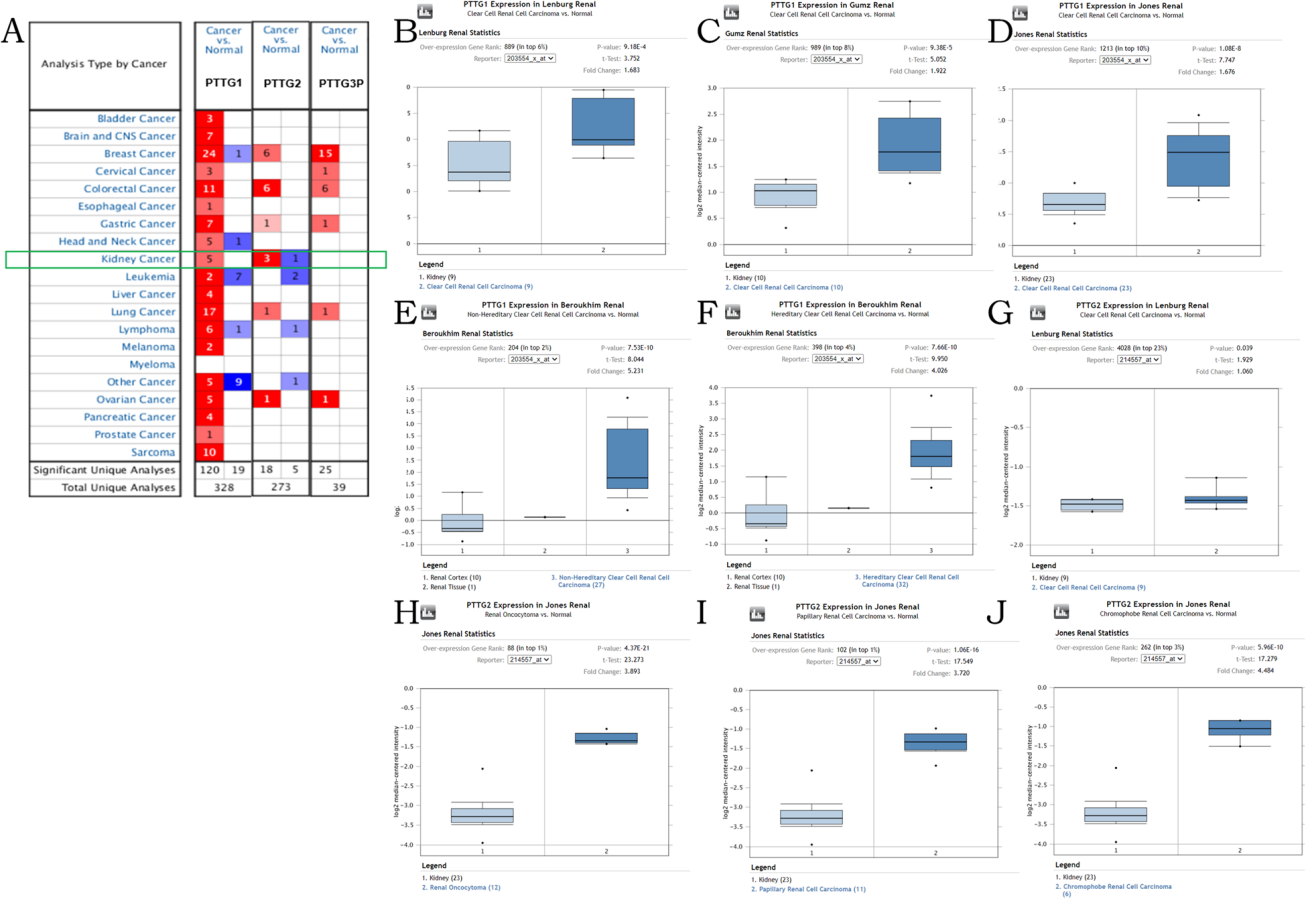
Transcriptional expression of PTTGs in 20 different types of cancer diseases (**a**). Difference of transcriptional expression was compared by students’ *t* test. Cut-off of *p* value and fold change were as follows: *p* value, 0.01; fold change, 1.5; gene rank, 10%; data type, mRNA. Significant changes of PTTGs expression in transcription level between KIRC and normal tissues (ONCOMINE) (**b**-**j**)

**Fig. 2:**
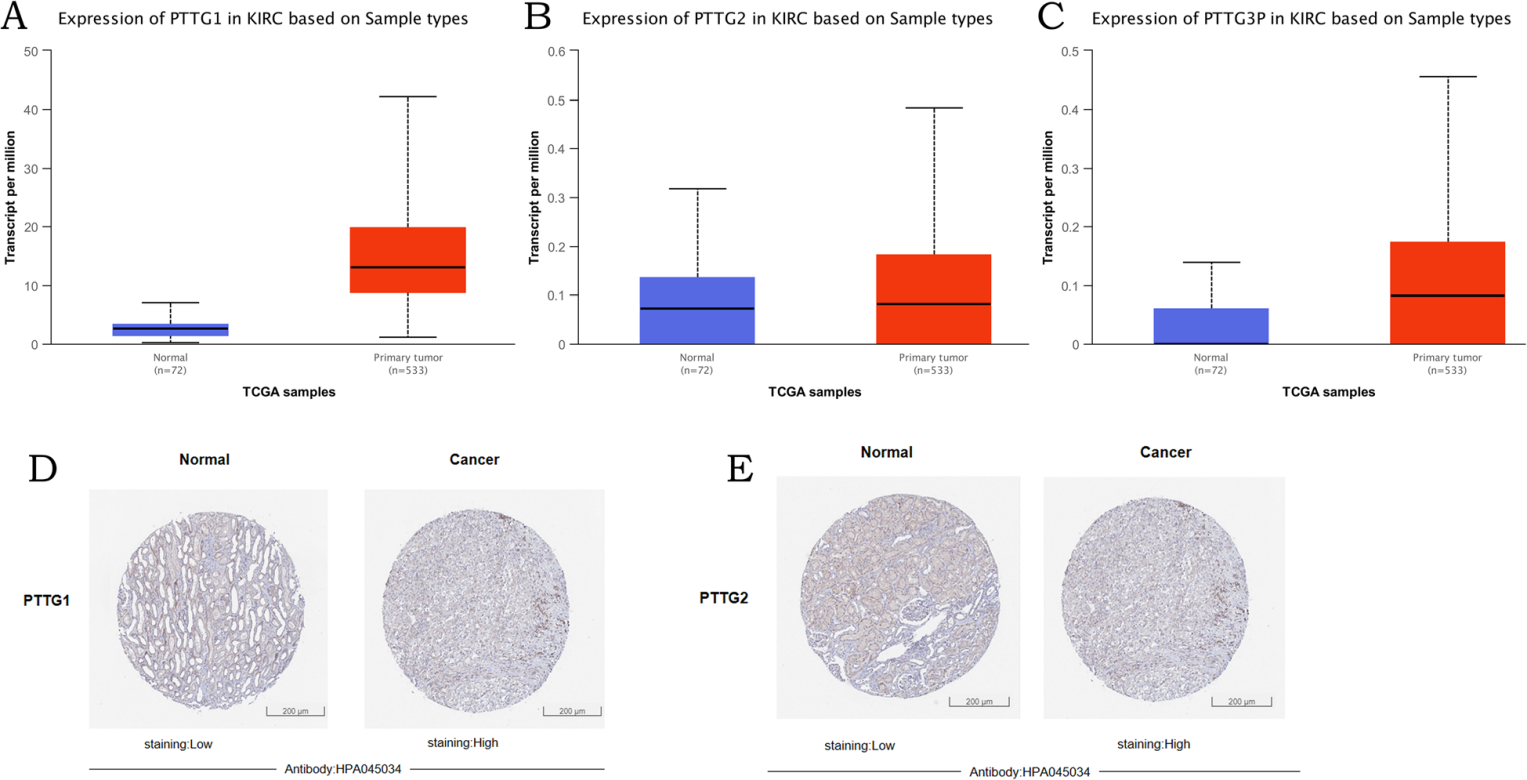
mRNA expression of distinct PTTG family members in KIRC and adjacent normal tissues (UALCAN). mRNA expressions of PTTG family members were found to be over-expressed in primary KIRC tissues compared with normal samples (**a**-**c**). *p<0.05, **p<0.01, ***p<0.001. Representative immunohistochemistry images of distinct PTTG family members in KIRC tissues and normal tissues (Human Protein Atlas), PTTG3P protein were not expressed in normal renal tissues, while PTTG1 and PTTG2 high and medium expressions were observed in KIRC tissues (**d**, **e**)

To explore distinct prognostic and potential therapeutic value of different PTTGs in kidney renal clear cell carcinoma patients, mRNA expression and protein expression were analyzed by ONCOMINE database, UALCAN, and Human Protein Atlas. As shown in Figs. 1 and 2, mRNA expressions of PTTG family members in 20 types of cancers were first measured and compared to normal tissues by ONCOMINE database. In Fig. 1, significant changes in *PTTG1* and *PTTG2* transcriptional levels between kidney renal clear cell carcinoma and normal tissues were observed in different datasets. In Beroukhim Renal Statistics, *PTTG1* over-expression was found in non-hereditary clear cell renal cell carcinoma tissues compared with normal tissues with a fold change of 5.231 (*p*=7.53E−10). Hereditary clear cell renal cell carcinoma was a fold change of 4.026 (*p*=7.66E−10). In Lenburg Renal Statistics, 1.683-fold change was found in *PTTG1* mRNA expression in kidney renal clear cell carcinoma tissues (*p*=9.18E−4). In Gumz Renal Statistics, they found 1.922-fold change in *PTTG1* mRNA expression in kidney renal clear cell carcinoma tissues (*p*=9.38E−5) while in Jones Renal Statistics, 1.676-fold change (*p*=1.08E−8) had been found. Significant upregulation of *PTTG2* was also found in kidney renal clear cell carcinoma tissues compared with normal tissues. The result from Lenburg Renal Statistics showed that there were 1.060-fold change (*p*=0.039) increased in *PTTG2* mRNA expression in kidney renal clear cell carcinoma tissues, respectively. The mRNA expression profiles of PTTG family members were furtherly examined by using UALCAN, which, unlike ONCOMINE, was sourced from clinical data on 31 cancer types in the Level 3 RNA-seq and TCGA databases. As shown in Fig. 2, mRNA expression of PTTGs members were all found to be significantly upregulated in kidney renal clear cell carcinoma tissues compared with normal samples (all *p*<0.05). To explore the protein expression patterns of PTTGs in kidney renal clear cell carcinoma, the Human Protein Atlas was applied. It has been found that *PTTG3P* protein was not expressed both in normal renal tissues and in kidney renal clear cell carcinoma tissues. Low protein expressions of *PTTG1*, *PTTG2* were expressed in normal renal tissues, while high protein expressions of them were observed in kidney renal clear cell carcinoma tissues (Fig. 2). In conclusion, the results showed that the transcriptional and proteinic expressions of PTTGs were over-expressed in patients with kidney renal clear cell carcinoma.

### Relationship between mRNA expression of genes of different PTTG family members and clinicopathological parameters in kidney renal clear cell carcinoma patients

**Fig. 3:**
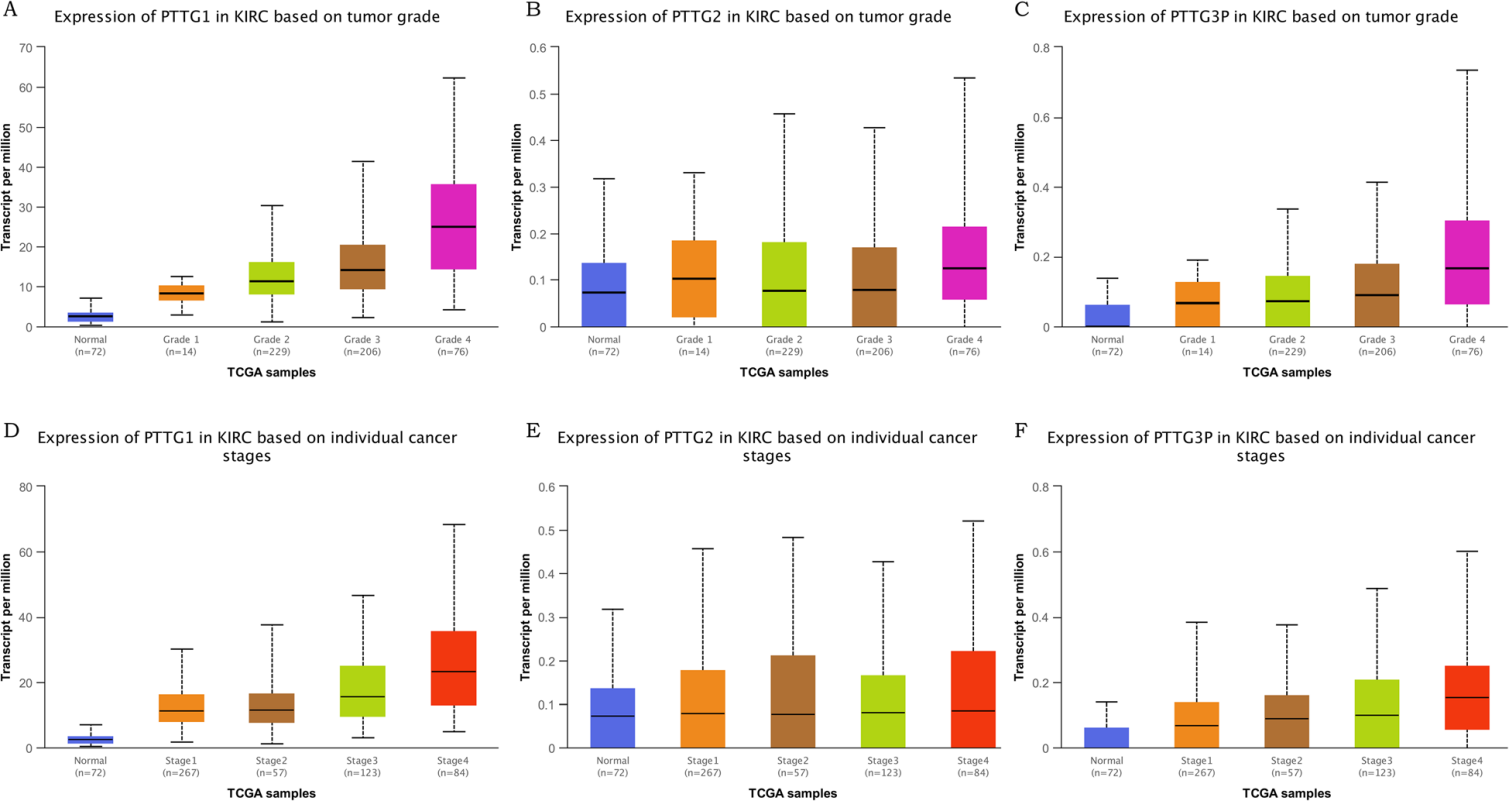
Association of mRNA expression of distinct PTTG family members with tumor grades of KIRC patients. mRNA expressions of PTTG family members were significantly related to tumor grades, and as tumor grade increased, the mRNA expressions of PTTGs tended to be higher, individual cancer stages similarly (**a**-**f**) *p<0.05, **p<0.01, ***p<0.001

After the discovery of the fact that mRNA expression and protein expressions were over-expressed in kidney renal clear cell carcinoma patients, the analysis of the relationship between mRNA expression of different PTTG family members with clinicopathological parameters of kidney renal clear cell carcinoma patients by UALCAN, including individual cancer stages and tumor grades, was conducted. As shown in Fig. 3, the mRNA expression of PTTG family members was correlated with individual cancer stage, indicating that patients with more advanced cancer stages tended to have higher PTTGs mRNA expression. Similarly, mRNA expressions of PTTG family members were significantly related to tumor grades, and as tumor grade increased, the mRNA expression of PTTGs had a tendency to increase. The results above suggested that mRNA expressions of PTTG family members were significantly associated with clinicopathological parameters in kidney renal clear cell carcinoma patients.

### Prognostic value of mRNA and protein expression of PTTGs in kidney renal clear cell carcinoma patients

**Fig. 4:**
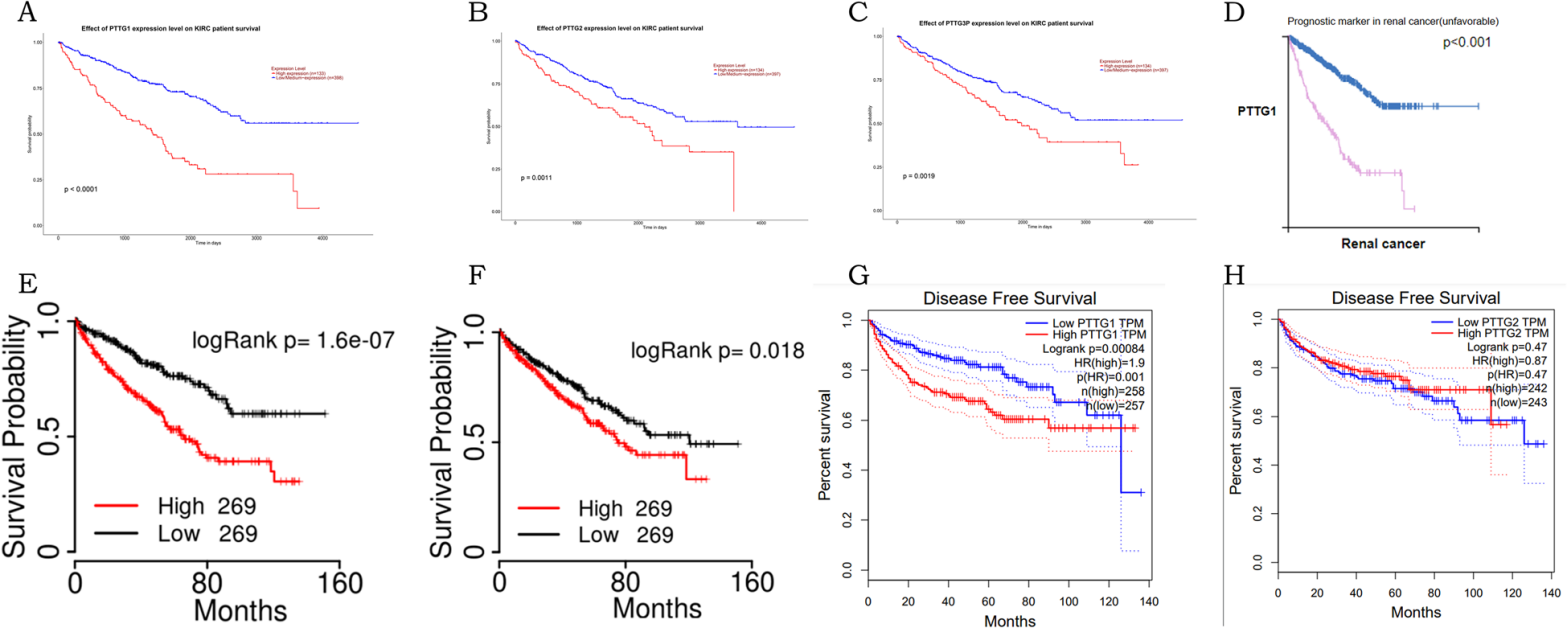
Prognostic value of mRNA expression of distinct PTTG family members in KIRC patients (UALCAN) (**a**-**c**). Prognostic value of protein expression of distinct PTTG family members in KIRC patients (Human Protein Atlas) (**d**). Prognostic value of mRNA expression of PTTG1, PTTG2 in KIRC patients (TCGAportal) (**e**, **f**). Prognostic value of mRNA expression of distinct PTTG family members in KIRC patients (GEPIA) (**g**)

UALCAN was used to analyze the prognostic values of the mRNA expression of PTTGs in kidney renal clear cell carcinoma patients. As shown in Fig. 4, mRNA expression in members of the PTTG family was significantly associated with prognosis in patients with kidney renal clear cell carcinoma disease. Firstly, the relationship among mRNA expression of different family members and prognosis of patients with kidney renal clear cell carcinoma disease was analyzed. Higher mRNA expression of PTTGs was significantly associated with shorter OS and disease-free survival of kidney renal clear cell carcinoma patients. Then Human Protein Atlas was used to analyze the prognostic values of the protein expression of PTTGs in kidney renal clear cell carcinoma patients. The results showed that the expression level of PTTGs was significantly correlated with prognosis in kidney renal clear cell carcinoma. Finally, TCGA portal was used to analyze the prognostic values of the mRNA expression of PTTGs in kidney renal clear cell carcinoma patients. The results also showed a correlation with prognosis. These results indicated that PTTGs mRNA and protein expression were significantly associated with prognosis in patients with kidney renal clear cell carcinoma and could be used as a biomarker to predict survival in patients with kidney renal clear cell carcinoma.

### The correlations between genetic mutations in PTTG family members and OS of kidney renal clear cell carcinoma patients

**Fig. 5:**
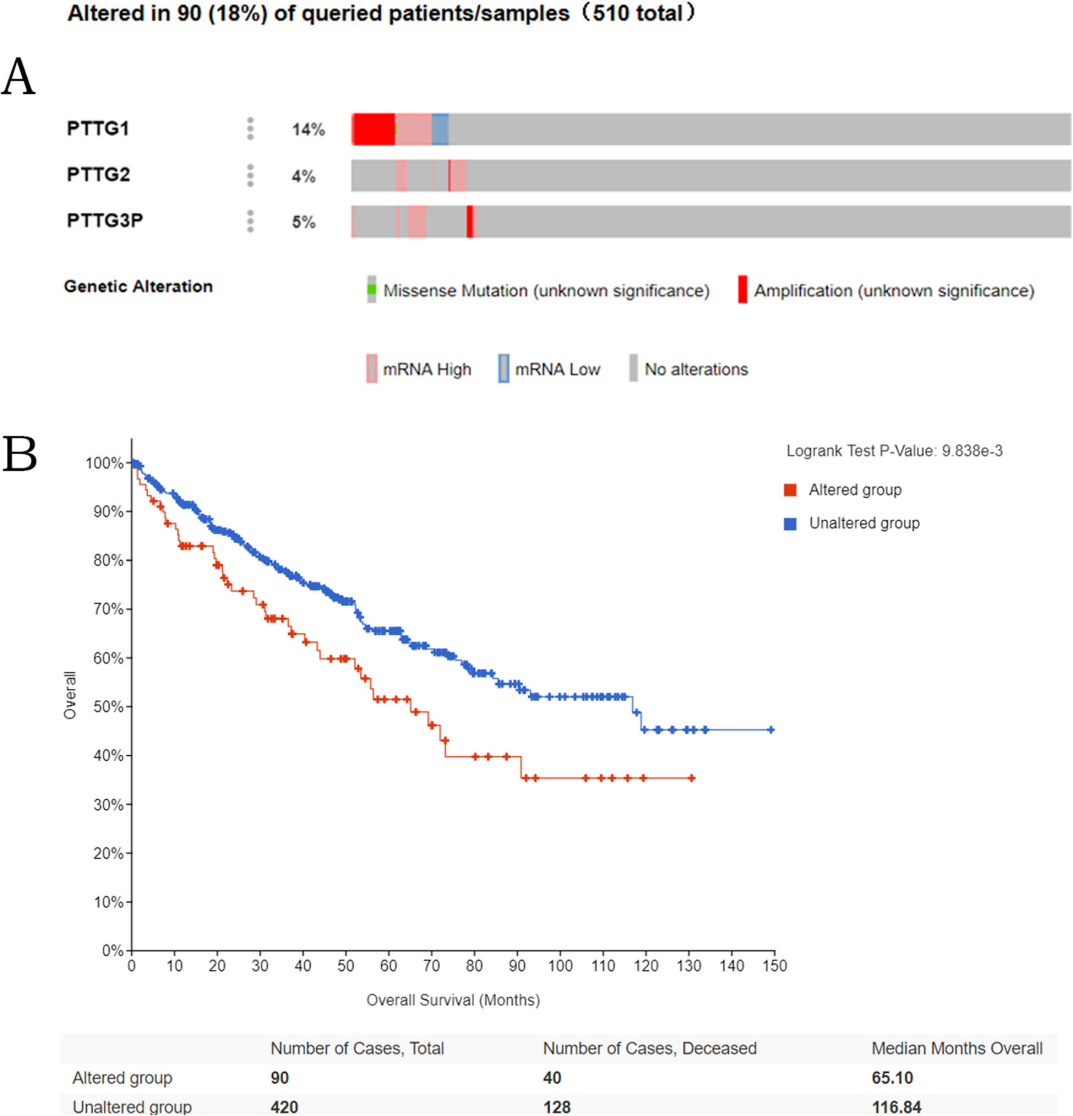
Genetic mutation rates of PTTG family genes in KIRC patients of KIRC patients (cBioPortal). Mutation rate (18%) of PTTGs was observed in KIRC patients, their mutation rates were 14%, 4%, and 5% respectively (**a**). Genetic alterations in PTTGs were associated with shorter OS of KIRC patients (**b**)

cBioPortal was used to analyze the genetic mutations of differentially expressed PTTG family members in kidney renal clear cell carcinoma patients. Based on Fig. 5, the mutation rate of *PTTG1*, *PTTG2*, and *PTTG3P* genes was 14%, 4%, and 5% respectively in 510 samples. Furthermore, the association between genetic mutations and the prognosis of kidney renal clear cell carcinoma patients was explored. And a statistically significant correlation was found between genetic mutations of PTTG family numbers and OS (*p*=9.838 E−3) in kidney renal clear cell carcinoma patients.

### PTTG1 and UBE2C are co-expressed in kidney renal clear cell carcinoma

**Fig. 6:**
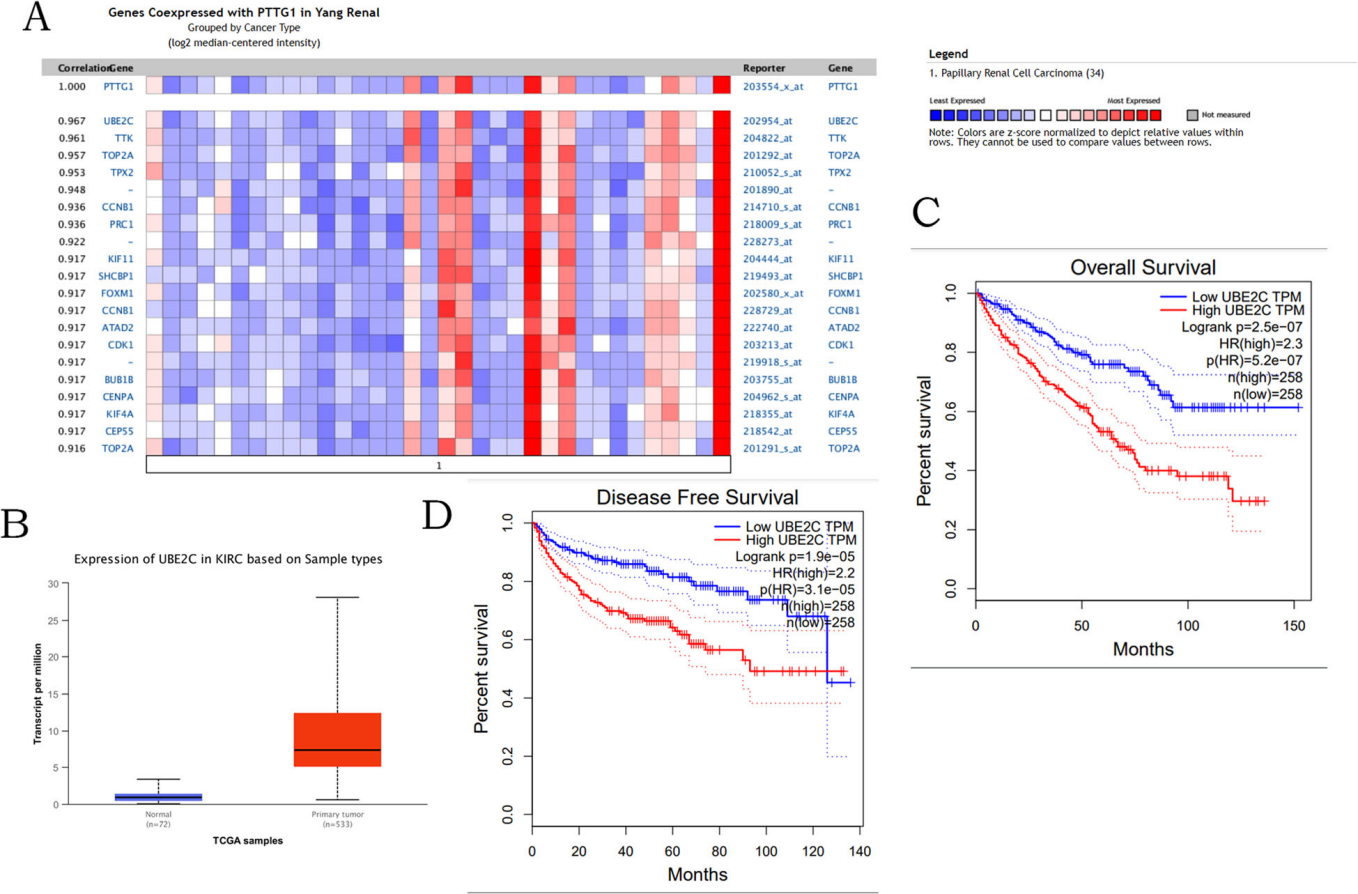
Co-expression profile of PTTG1 identified (ONCOMINE) (**a**) mRNA expression of UBE2C in KIRC samples and adjacent normal renal samples (GEPIA) (**b**). The prognostic value for the expression of UBE2C (GEPIA) (**c**, **d**)

In order to further study the potential mechanism of PTTG1 in kidney renal clear cell carcinoma, the co-expression database was used to mine the co-expression data of PTTG1 (Fig. 6a). The data above indicated that PTTG1 may be associated with the UBE2C signaling pathways in kidney renal clear cell carcinoma.

### The validation and survival analysis of UBE2C in kidney renal clear cell carcinoma

The expression of UBE2C was validated in GEPIA and UNCLAN database. It had been found that UBE2C were significantly upregulated in the expression of UBE2C which were validated in GEPIA and UNCLAN database. UBE2C were significantly upregulated in kidney renal clear cell carcinoma (Fig. 6b). The survival analysis in GEPIA and UNCLAN database confirmed that the upregulation of UBE2C was correlated with shorter overall survival and disease-free survival of kidney renal clear cell carcinoma patients respectively (Fig. 6c-d).

### Networks analyses and functional enrichment analyses of PTTG family genes and their neighboring genes in kidney renal clear cell carcinoma patients

**Fig. 7:**
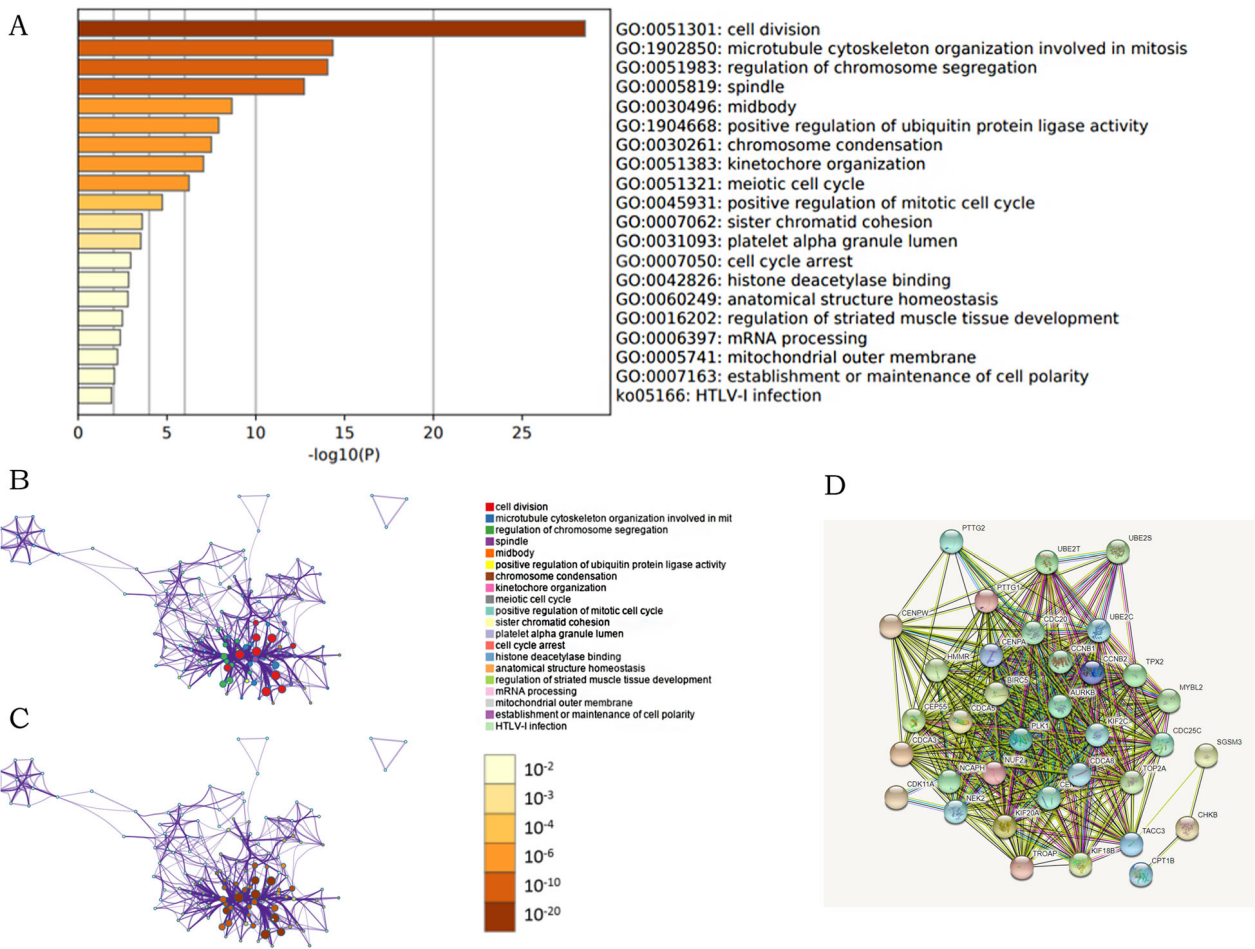
GO functional enrichment analysis predicted four main functions of PTTGs. Functionally similar gene including biological process, cellular components, molecular functions, and KEGG pathway analysis (**a**-**c**). PPI network was generated from members of the PTTG family genes and their similar genes (STRING) (**d**)

After confirming the correlation between genetic mutations in PTTG family numbers and prognose values, similar genes of the PTTG family (90 in total) obtained from GEPIA2 and Metascape were used for GO enrichment to explore the interaction between similar genes. Based on 90 adjacent genes, the online tools of Metascape and STRING were used for functional and pathway enrichment analysis, and a PPI interaction network was established to explore the biological classification of PTTG. The functions of PTTG family members neighboring genes were predicted by analyzing GO and KEGG in Metascape. The GO enrichment items were classified into four functional groups: KEGG pathway, biological process group, molecular function group, and cellular component group (Fig. 7). The PTTG family members and their similar genes were enriched in the following information, cell division, microtubule cytoskeleton organization involved in mitosis, regulation of chromosome segregation, positive regulation of ubiquitin protein ligase activity, chromosome condensation, kinetochore organization in biologic processes (BP), and spindle, midbody, platelet alpha granule lumen, mitochondrial outer membrane in cellular components (CC), and HTLV-I infection in KEGG pathway. The PPI network interactions of PTTG family genes and similar genes were conducted by String to seek possible downstream targets and mechanism research, and it was found that *CENPW*, *CENPA*, *HMMR*, *CDC20*, and other genes can be used as the target genes for further research and analysis.

## Discussion

As mentioned earlier, the PTTG family is widely expressed in a variety of tumors. Although PTTG has been shown playing a role in the occurrence and prognosis of many cancers, the role of PTTG in kidney renal clear cell carcinoma still requires further bioinformatics analysis. We hope that our study will provide new insights into the clinical diagnosis, therapeutic targets, and tumor development mechanisms of kidney renal clear cell carcinoma.

The results of the study indicate that overexpression of the PTTG gene is found in kidney renal clear cell carcinoma, and PTTG is significantly associated with individual cancer stage and tumor grade in kidney renal clear cell carcinoma patients. mRNA expressions of PTTGs were significantly associated with shorter OS in kidney renal clear cell carcinoma patients. *PTTG1* and *PTTG2* protein over-expressions were also associated with shorter OS. Moreover, the mutation rate (18%) of PTTGs was observed in kidney renal clear cell carcinoma patients and the genetic alteration in PTTGs was associated with shorter OS. Finally, the PTTG family members and their similar genes were enriched in cell division, microtubule cytoskeleton organization involved in mitosis, regulation of chromosome segregation, positive regulation of ubiquitin protein ligase activity, chromosome condensation, kinetochore organization in biologic processes (BP), and spindle, midbody, platelet alpha granule lumen, mitochondrial outer membrane in cellular components (CC), and HTLV-I infection in KEGG pathway.

*PTTG1* has been shown to induce oncogenesis by facilitating cell proliferation and independent tumorigenesis in vivo and in vitro studies [12]. *PTTG1* expression was significantly correlated with lymph node metastasis, clinical stage, and degree of tumor differentiation in patients with laryngeal cancer [21]. *PTTG1* was overexpressed in kidney renal clear cell carcinoma, the higher the grade of tumor cells, the higher the expression is, the poorer prognosis is associated [22]. In this study, consistent with previous studies, *PTTG1* mRNA and protein expression was found to be significantly higher in kidney renal clear cell carcinoma tissues compared with normal tissues, and *PTTG1* mRNA expression was significantly associated with individual cancer stage and tumor grade in patients. In addition, high *PTTG1* expression was also significantly associated with shorter survival in patients with kidney renal clear cell carcinoma, suggesting that *PTTG1* was involved in the development of kidney renal clear cell carcinoma tumors.

It has been found that *PTTG2* leads to the downregulation of E-cadherin and the increase of vimentin level, which is involved in the occurrence and development of tumors [23]. Additionally, the overexpression of *PTTG2* also inhibits apoptosis in glioblastoma by affecting caspase-3-dependent signaling pathways [24]. In this study, significantly higher mRNA and protein expression of *PTTG2* were found in kidney renal clear cell carcinoma tissues, and mRNA expression of *PTTG2* was remarkably correlated with patients’ individual cancer stages and tumor grades. Moreover, higher mRNA expression of *PTTG2* was also significantly related with poorer OS of kidney renal clear cell carcinoma patients and it was an independent prognostic factor for shorter OS of kidney renal clear cell carcinoma patients, playing an oncogenic role of *PTTG2* in kidney renal clear cell carcinoma.

Overexpression of *PTTG3P* has been shown to promote tumorigenesis by upregulating *PTTG1* and activating *PI3K/Akt* signaling and downstream signaling, including genes related to cell cycle progression, apoptosis, and epithelial mesenchymal transformation (EMT) [25]. Previous studies have also reported high expression of *PTTG3P* in breast cancer and it has been found that *PTTG3P* expression is inversely correlated with estrogen receptor (ER) and progesterone receptor (PR) status, and high expression of *PTTG3P* is associated with poor prognosis of breast cancer [26]. Some studies have shown that enhanced *PTTG3P* expression stimulates the migration and invasion of ESCC cells, thus promoting the expression levels of *PTTG1* and *PTTG2* in vitro, and realizing its carcinogenic function by positively regulating its parent genes *PTTG1* and *PTTG2* [14]. In this study, higher *PTTG3P* expression was found in the kidney renal clear cell carcinoma tissues, and *PTTG3P* expression was significantly correlated with individual cancer stage and tumor grade of the patients. High expression of *PTTG3P* was also significantly associated with adverse OS in patients with kidney renal clear cell carcinoma and it was an independent prognostic factor for shorter OS in patients with kidney renal clear cell carcinoma, suggesting that *PTTG3P* is involved in the oncogenesis of kidney renal clear cell carcinoma. UBE2C is an ubiquitin-conjugating enzyme which acts as the ubiquitin-activating enzyme E1 and ubiquitin protein ligase E3 to catalyze the degradation of proteins into smaller polypeptides, amino acids, and ubiquitin in the 26S proteasome. UBE2C participates in carcinogenesis by regulating the cell cycle, apoptosis, and transcriptional processes. UBE2C upregulation has been found correlated with poor overall survival (OS) and progression-free survival (PFS) in patients with NSCLC [27], and our study indicated that PTTG1 may be associated with the UBE2C signaling pathways in kidney renal clear cell carcinoma. The expression of UBE2C in kidney renal clear cell carcinoma is different and the difference is significant, which is important for survival.

## Conclusion

The PTTG family genes play an important role in kidney renal clear cell carcinoma. High expression of PTTG family genes may be a diagnostic and prognostic indicator in patients with kidney renal clear cell carcinoma. In addition, the relationships between genes related to cell division and kidney renal clear cell carcinoma disease as well as the relationship between genes such as *CENPW*, *CENPA*, *HMMR*, and *CDC20*, and kidney renal clear cell carcinoma are also worthy of further study.

### Uniqueness and limitations

Data in these databases is real and reliable, the useful genes can be quickly screened out. Moreover, the multi-database verification with different data sources can provide theoretical guidance for the next clinical experiment, what’s more, the PTTG family as a whole has never been reported as a prognostic parameter for renal clear cell carcinoma. But there are some limitations that need to be recognized in the current study. Firstly, our data came from online public database, so the potential diagnostic and therapeutic values of PTTG family genes were not confirmed in kidney renal clear cell carcinoma patients because there are no lab reports, and our results should be confirmed by further studies.

## Data Availability

The data used to support the findings of this study are included within the article.

## Abbreviations

KIRC: Kidney renal clear cell carcinoma
